# Lightweight remote sensing change detection with progressive multi scale difference aggregation

**DOI:** 10.1038/s41598-025-10972-5

**Published:** 2025-08-18

**Authors:** Yinghua Fu, Haifeng Peng, Tingting Zhao, Yize Li, Jiansheng Peng, Dawei Zhang

**Affiliations:** 1 School of Optical-Electrical and Computer Engineering, University of Shanghai for Science and Technology, Shanghai, 200093 China; 2 Department of Artificial Intelligence and Manufacturing, Hechi University, Hechi, 546300 China

**Keywords:** Urban ecology, Ecology

## Abstract

Change detection (CD) is the process of acquiring changes in the ground surface by analyzing remotely sensed images of the same location at two different stages. Deep learning is becoming increasingly popular for change detection tasks in remote sensing due to its significant advantages in deep feature representation and nonlinear problem modeling. However, many previous neural network-based approaches require a large number of parameters and computations and high-performance hardware, which makes their practical application in remote sensing challenging. Some lightweight change detection techniques are implemented by enhancing deep features and then extracting their difference information, which tends to ignore many shallow features. We propose a lightweight network MobileNetV2 as an encoder and a modified UNet as a decoder, where MobileNetV2 extracts features from bit-time images and UNet performs layer-by-layer fusion of different stages of difference images to enhance the representativeness of changes. Since MobileNetV2 is used as the encoder of UNet, the computation of the proposed method is greatly reduced. Experiments show that our method has the lowest computational cost (2.38G) and the fewest parameters (2.95M) compared to the current mainstream lightweight networks (FC-Siam-diff32, FC-Siam-conc, A2Net and BIT). Three remote sensing public datasets, SYSU-CD, BCDD and LEVIR-CD, are introduced to validate the proposed method in this paper, and the results show that the F1 of the method in this paper reaches 82.84%, 94.51% and 90.89% respectively. All code as well as detailed instructions are available at https://github.com/tawneydaylily/Mobile-CDNet.

## Introduction

Remote Sensing Change Detection (RSCD) is based on the observation of the same object or phenomenon at different times to determine their difference^1^. At present, researchers have done a lot of works on the application of change detection technology from different perspectives such as urban change analysis^2^, disaster monitoring^3^, land management^4^ and so on. Detecting the change of land cover provides important data support for geological survey^5^. RSCD is still full of challenge due to the complexity of ground objects, variety of seasons and the influence of the illumination.

Change detection (CD) has been developed in recent decades^6^. Traditional CD methods rely on the expertise and manual-crafted features. Traditional CD methods can achieve high recognition accuracy on small-scale data sets, but they cannot decrease “same object with different spectrum” and “different object with same spectrum”^7^ and the higher-level scene context semantic features in images cannot be fully exploited and utilized by hand-designed features. Deep learning has garnered extensive attention in computer vision research. Deep learning-based RSCD has risen in recent years, enabling automatic feature representation extraction from large-scale datasets. Deep learning facilitates more effective utilization of advanced contextual semantic features in images, thereby enhancing the accuracy of CD. Various deep learning models, including Deep Belief Networks (DBN)^8^, Stacked Autoencoders (SAE)^9^, Generative Adversarial Networks (GAN)^10^, Recurrent Neural Networks (RNN)^11^, and Convolutional Neural Networks (CNN)^12^, are widely applied in RSCD^13^. These models leverage diverse architectures to address complex spatial and temporal data in CD tasks, achieving high detection accuracy but often requiring high computational demands and extensive training datasets. For example, DBN and GAN excel in complex scenarios yet are computationally intensive, whereas CNNs offer efficient spatial feature extraction but may be less effective for temporal dependencies compared to RNNs. These techniques substantially enhance the ability of traditional methods to address complex challenges, such as “same object, different spectrum” and “different objects, same spectrum,” while their reliance on extensive computational resources and large datasets limits their applicability in resource-constrained settings. Despite these advances, recent RSCD methods, while achieving promising detection performance, often rely on computationally intensive models with numerous parameters. For example, the DSIFN method requires 50.71 M parameters and 164.56 G FLOPs to achieve an F1-score of 0.9135 on the LEVIR dataset. Similarly, SNUNet requires 27.07 M parameters and 246.22 G FLOPs to attain an F1-score of 0.9003 on the BCDD dataset. Such resource-intensive models are impractical for deploying RSCD methods on platforms like unmanned aerial vehicles (UAVs) or satellites, where computational and energy resources are limited. This highlights the critical need for a lightweight RSCD method with fewer parameters, lower computational complexity, and robust performance.

At present, lightweight networks are starting to emerge in the field of RSCD, which easily cope with large-scale remote sensing data and automatically extract features with their low memory footprint and fast processing capabilities^14^. There are three perspectives on the model lightweighting and over-parameterization for the researchers to consider^15^: network architecture, network optimization, and hardware acceleration. Lightweighting deep learning architecture can be achieved through model pruning^16,17^, knowledge distillation^15^, and developing the smaller models^18^. Nowadays, the lightweighting of deep learning architectures is mainly achieved by developing smaller models that involves either innovating convolution blocks or reducing the width and depth of the tranditional convolution operations. Architectures with innovative convolution are widely used in RSCD such as ShuffleNetV2^19^, SqueezeNet^20^ and GhostNet^21^, which are optimised for the high computational efficiency and lower latency requirements in RSCD. RegNet^22^ and MicroNet^23^ were designed for model lightweight by reducing the width and depth of convolution operations, which provide a high degree of configurability and flexibility. Meanwhile, the lightweighting of models has also led to the emergence of new architectures, such as EfficientNet-Lite and MobileViT^24^, which demonstrate compact structures and good accuracy in many vision tasks. However, despite the significant progress these new lightweight models have made in vision tasks, according to tests from Google and other public sources, their inference speed per image is slightly slower than that of MobileNetV2. MobileNetV2^25^ as a new developed architecture with innovating convolution blocks uses depthwise separable convolutions to decompose standard convolutions into depthwise and point-by-point convolutions to significantly reduce computation and parameter count, where linear bottleneck layers and inverted residuals are used to enhance feature representation. Among these popular lightweight CNN network, MobileNetV2 offers an optimal balance between parameter count and model accuracy.

In this paper, we develop a novel lightweight architecture, Mobile-CDNet, for RSCD, which takes the enhanced MobileNetV2 as the backbone and optimizes U-Net with depthwise separable convolutions and multi-level difference image features as well as difference fusion model in the decoder, and a simple classifier is used to obtian the accurate changes. The main contributions of our work can be summarized as follows: (1) We propose the Mobile-CDNet model, involving innovating convolution block to improve computational efficiency and significantly reduce floating-point operations (FLOP); (2) We effectively integrate multi-level difference image features in the decoder to ensure robust performance from coarse to fine processing stages; (3) Our difference fusion module consists of ordinary convolutional layers and pooling layers with additional max-pooling to alleviate noise, extracting differential information from backbone network features and enhancing the learning of useful information; (4) We get accurate change area by introducing sigmoid function to recognize the change. Experimental validation on three public CD datasets demonstrates the effectiveness of Mobile-CDNet compared to existing methods that Mobile-CDNet excels in both computational efficiency and detection performance. By reducing computational costs without sacrificing accuracy, Mobile-CDNet provides a practical solution for resource-limited remote sensing applications. These findings emphasize its practical deployment potential across various real-world application scenarios.

## Related works

In this section, we review related work in traditional learning methods, deep learning method and lightweight architecture.

### Traditional learning methods

PBCD contains three categories, two of which are widely used: algebra based techniques and transformation based techniques. Algebra based techniques includes image difference^26^, image ratio^27^, change vector analysis (CVA)^28^. Image difference involves subtracting one image from another to obtain a difference image which then are threasholded to get change regions. Image ratio is similar to image difference with ratio instead of subtraction. CVA is to obtain the change vector to represent the change of surface features by calculating the difference of each band of the multi-temporal remote sensing image. The detection performance of algebra based techniques is sensitive to random noise because of the point-to-point operation.

The transformation based techniques are designed to mine the discriminative characteristics of the data by re-selecting and combining different channels of the image or projecting it to the subspace, and use more representative feature to improve the accuracy and efficiency of change detection, which includes Principal Component Analysis (PCA)^29–33^, Multivariate Alteration Detection (MAD)^34,35^ and Slow Feature Analysis (KSFA)^36^. PCA presupposes linear correlations among spectral bands, which may not fit for nonlinear applications especially for striving to perceive intricate or subtle changes. MAD is susceptible to noise and involves a substantial amount of computational calculations.

OBCD identifies changes by segmenting the image into disjoint and homogeneous objects, and comparing and analyzing the dual-temporal-phase objects. Wu et al.^37^ proposed a post-classification change detection method based on iterative slow feature analysis and Bayesian soft fusion. But OBCD is significantly impacted by segmentation errors and relies heavily on initial setup.

### Deep learning methods

DBNs learn hierarchical feature representations from multi-temporal remote sensing images through pre-training and fine-tuning. Liu et al.^38^ proposed an unsupervised multi-temporal spectral demixing method based on DBN for detecting multiple changes in hyperspectral images. Samadi et al.^39^ proposed a DBN-based change detection method for SAR images using a new morphological image-based training method, from basic DBN model construction to complex models incorporating the latest deep learning techniques, DBN has demonstrated a strong capability in processing high-dimensional remote sensing data but requires significant computational resources and time.

SAEs extract and reconstruct features by learning compact representations of input data using encoder-decoder structures. Farahani et al.^40^ performed feature fusion of SAR and optical images using an autoencoder-based approach to improve change detection accuracy. Saha et al.^41^ proposed an unsupervised LSTM-based method to solve the problem of change detection in time series. Shu et al.^42^ proposed a patch-based change detection method to convert irregularly shaped change labels into regular maps through a mask function, enabling the network to end-to-end learn patches. Qu et al.^43^ introduced a unique DDNet that merges spatial and frequency domain elements to improve classification performance. However, SAE relies heavily on the quality and diversity of training data.

GANs generate synthetic images suitable for CD by utilizing generator and discriminator to compensate for insufficient labeled data. Gong et al.^44^ proposed a GAN-based model to restore the distribution of the training data from noise in multispectral images. The difference maps obtained by traditional methods are input as training data to the generator which will learn the mapping between the distribution of training data and their labels through adversarial interaction with the discriminator to obtain the accurate difference maps, but the training process may be unstable and requires complex hyperparameterization and training strategies.

RNNs capture temporal changes in multi-temporal remote sensing images by using recurrent structures and Long Short Term Memory (LSTM) units. Lyu et al.^45^ developed an end-to-end RNN to solve multispectral/hyperspectral image change detection tasks, where a LSTM-based RNN is taken to learn a joint spectral-temporal feature representation from bitemporal image sequences. Mou et al.^46^ proposed a new end-to-end RNN architecture for change detection in multispectral images through learning a joint spatial-spectral-temporal feature representation, which involves the extra spatial information. RNN with LSTM block is hard to become a lightweight model because of intrinsic big parameters.

CNNs effectively extract multi-scale spatial features through convolutional kernels and pooling layers for RSCD. Daudt et al.^47^ proposed three different end-to-end change detection architectures, namely FC-early fusion, FC-Siam-conc and FC-Siam-diff, the difference of which lies in the bitemporal image fusion strategy and jump connection options. Inspired by UNet++^48^, Fang et al.^49^ designed a densely connected siamese network SNUNet-CD, which alleviates the loss of deep feature localization information by aggregating features at multi-level semantic levels. Peng et al.^50^ proposed a unet++-based early fusion architecture to obtain multiscale features through dense skip connections. Bi-temporal Image Transformer (BIT)^51^ introduced the transformer block into RSCD for the first time by mapping high-level feature as semantic tokens and capturing contextual information within space-time relation, which facilitates the identification of interesting changing features while excluding irrelevant spurious changing features. The ChangeFormer^52^ is a purely transformer-based change detection model which utilizes MiT^53^ as the backbone and performed well in semantic segmentation models for the change detection task. Here, a hierarchical transformer encoder is integrated with a multi-layer perceptual decoder to efficiently extract the required multi-scale long-range dependence. Hao et al.^54^ introduce dynamic convolution block to cope with change regions of different scales to enhance the expression ability of the model while exploiting multiscale image features. But many CNN models contain millions or even billions of parameters, which is contrary to the goal of lightweight models.

### Lightweigt architectures

Lightweight neural networks are gradually showing the potential to replace common architectures such as traditional VGG, ResNet and DenseNet in the RSCD. Ma et al.^19^ proposed that ShuffleNetV2 reduces computational complexity and improves feature fusion through channel grouping and channel shuffle, which requires extensive experimentation during model optimization and hyperparameter tuning to find the best configuration. Iandola et al.^20^ proposed SqueezeNet achieving fast change detection with a simplified feature extraction module, but the performance was limited in complex scenes. GhostNet^21^ proposed by Huawei company takes a dual-channel bottleneck structure demonstrating the effectiveness of ghost features lightweight deformation of preceding layer features. RegNet ensures that the network can extract features efficiently at different scales by regularizing the design space, but its design and optimization process is a little complex. Li et al.^23^ proposed MicroNet decomposing the depthwise convolutions into a combination of two depthwise separable convolutions and innovatively taking the DY-Shift-Max activation function to improve the performance of MobileNet when FLOPs are below 25M. The introduction of these concepts has expedited the development of lightweight modeling in RSCD.

Recent studies have introduced novel technical approaches to complement these advancements. Song et al.^55^ proposed SNUNet-CD a densely connected twin-network leveraging residual learning and attention mechanisms for efficient and accurate change detection through feature extraction and comparative learning from the input images. Luo et al.^56^ introduced LWCDnet a lightweight cloud detection method tailored for efficient cloud region detection in remote sensing imagery. Huang et al.^57^ presented RSR-Net, a lightweight network structure integrating residual blocks and group convolution, incorporating Squeeze-and-Excitation (SE) modules as foundational units for the encoding tasks. Additionally, Sun et al.^58^ devised LRSR-net to mitigate feature loss involved by pooling layers, which enables the network to learn road features effectively with fewer parameters. These models refine feature extraction through residual learning and attention mechanisms to enhance detection accuracy without compromising efficiency, which allow deployment on resource-limited remote sensing scenarios.

## Methods

In this section, we first give an overview of the proposed method and then describe the proposed encoder and decoder in detail, finally, we will present the loss fusion.

### Network architecture

**Fig. 1:**
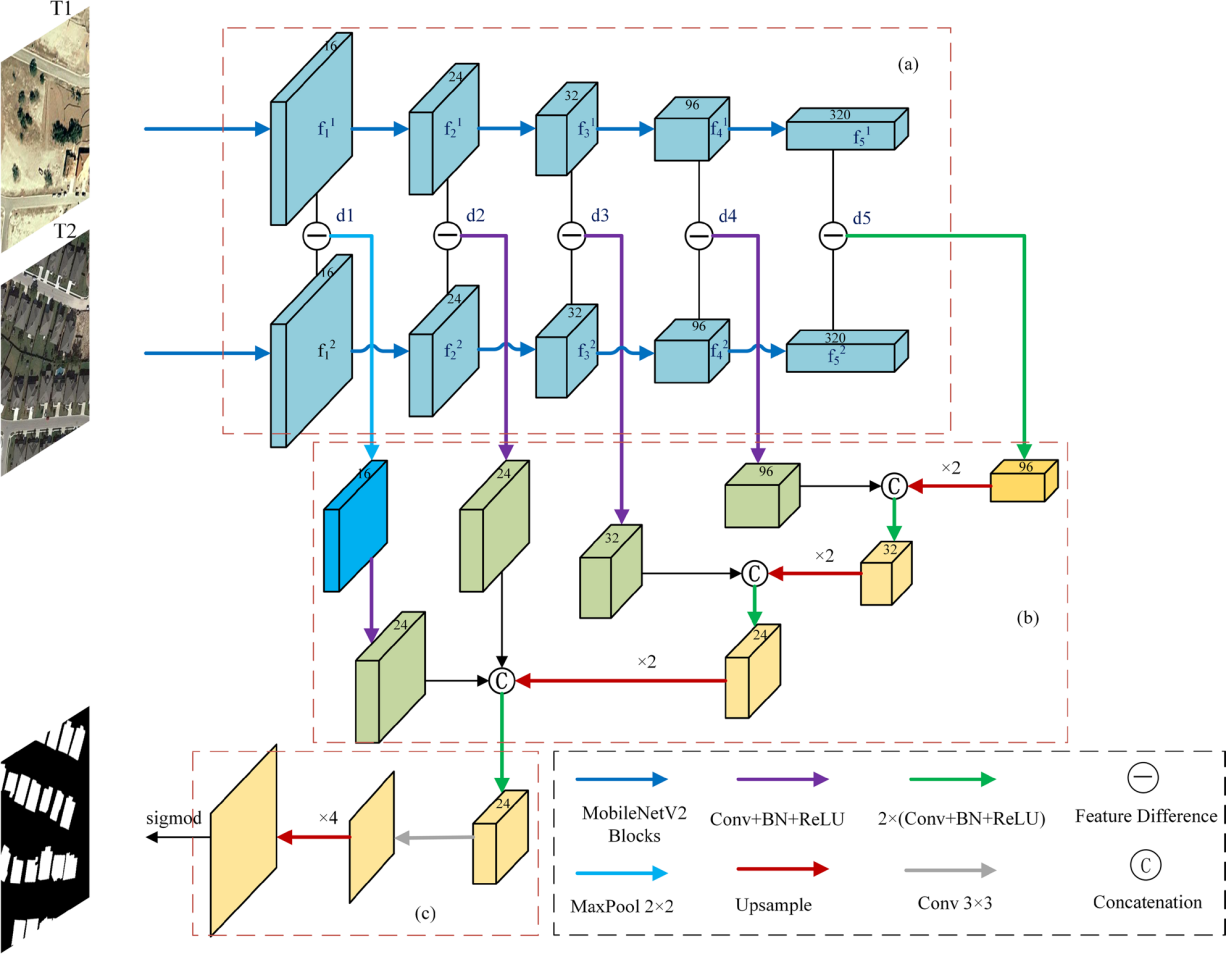
Overall structure of the Mobile-CDNet. (**a**) A dual-stream encoder with shared weights. (**b**) A decoder with multiscale change in feature maps via skip connections. (**c**) The prediction head.

Mobile-CDNet proposed in this article is an end-to-end dual-stream deep learning architecture illustrated in Fig. 1, which consists of three components: encoder, decoder and classifier, and the structure of later fusion^59^ is taken.

In the encoding stage, the bitemporal remote sensing images T1 and T2 are the input of the Mobile-CDNet. A dual-stream encoder with shared weights illustrated in Fig. 1a, MobileNetV2, is designed to extract discriminative features. To adapt the (RSCD) task, the backbone of MobileNetV2 is modified, and the global average pooling layer and the last fully connected layer are removed from the original architecture, which allows the model to better capture and represent the distinctive featuers of the input images in relation to change detection.

MobileNetV2 incorporates a convolutional layer with a stride of 2 in each stage. Hence the feature maps from each stage are downsampled to the one with a half of resolution compared to the previous stage. This downsampling enables the network to progressively extract the high-level semantic information while reducing the spatial dimensionality of the feature maps.

The bitemporal features from five stages can be denoted as $$ f_1^1 ,$$ $$ f_1^2, $$ $$\ldots $$, …, $$ f_5^2 $$ respectively, and then the temporal difference of the features for each stage are obtained through an elementwise subtraction operation and a subsequent absolution operation and denoted as $$ d_1 $$, …, $$ d_5 .$$ The formula is given in Eq. (1) from bitemporal features.

1 $$\begin{aligned} d_i = |f_i^1 \ominus f_i^2|, i \in 1,2,3,4,5 \end{aligned}$$

where $\ominus$ means elementwise subtraction operation. By employing this encoding strategy, Mobile-CDNet effectively captures essential features from the bitemporal remote sensing images T1 and T2, facilitating subsequent change detection processes.

In the decoding stage, a skip connection structure similar to UNet is introduced to sequentially feed the multiscale change feature maps into the decoder to obtain the change feature maps, as depicted in Fig. 1b.

In the pixel-wised classification stage, the input F $$ \in R^{\frac{H_0}{4}\times \frac{W_0}{4} \times C } $$ is from the last layer decoder, where $$H_0 $$ and $$W_0$$ are the height and width of the original image respectively. The prediction head (Fig. 1c) is to generate the predicted change probability map P $$ \in R^{ H_0\times W_0 \times C }, $$ which is given by Eq. (2).

2 $$\begin{aligned} P = sigmoid((Bilinear(Conv_{3*3}(F))) \end{aligned}$$

where $Conv_{3*3}$: $R^{\frac{H_0}{4}\times \frac{W_0}{4} \times C }$ $\rightarrow$ $R^{ H_0\times W_0 \times C }$ is one $3\times 3$ convolutional layer, and Bilinear is an upsampling function with four times of the linear interpolation. Sigmoid is used to generate the 0-1 probability map of the changed region.

### Encoder

MobileNet^60^ introduced by Howard et al. incorporates depthwise separable convolution^61^, which plays a crucial role in reducing computations and increasing the processing speed. This property becomes highly significant particularly for deep learning models with numerous convolutional layers.

**Fig. 2:**
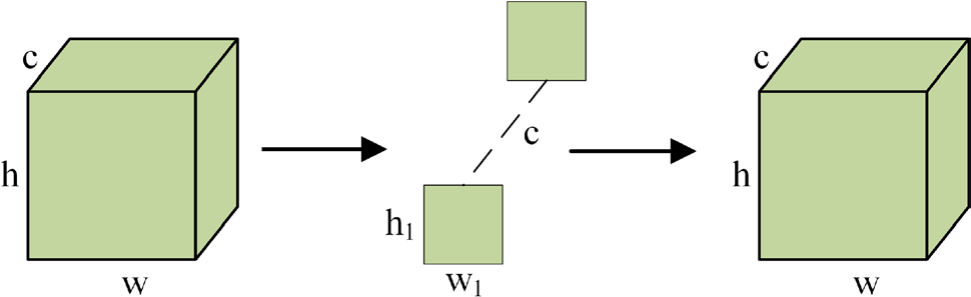
The structure of the depthwise convolution.

The convolution operation is split into two distinct operations in depthwise separable convolution: depthwise convolutions and pointwise convolutions. In the depthwise convolution step, as depicted in Fig. 2, each filter is applied individually to each channel of the input image. This process allows the model to capture spatial correlations within each channel while significantly reducing the parameters and computational cost compared to traditional convolutional layers.

Compared to MobileNetV1, MobileNetV2 is known for its improved speed by introducing two additional characteristics linear bottleneck layers and shortcut connections between the bottlenecks, which contributes to its enhanced performance.

**Fig. 3:**
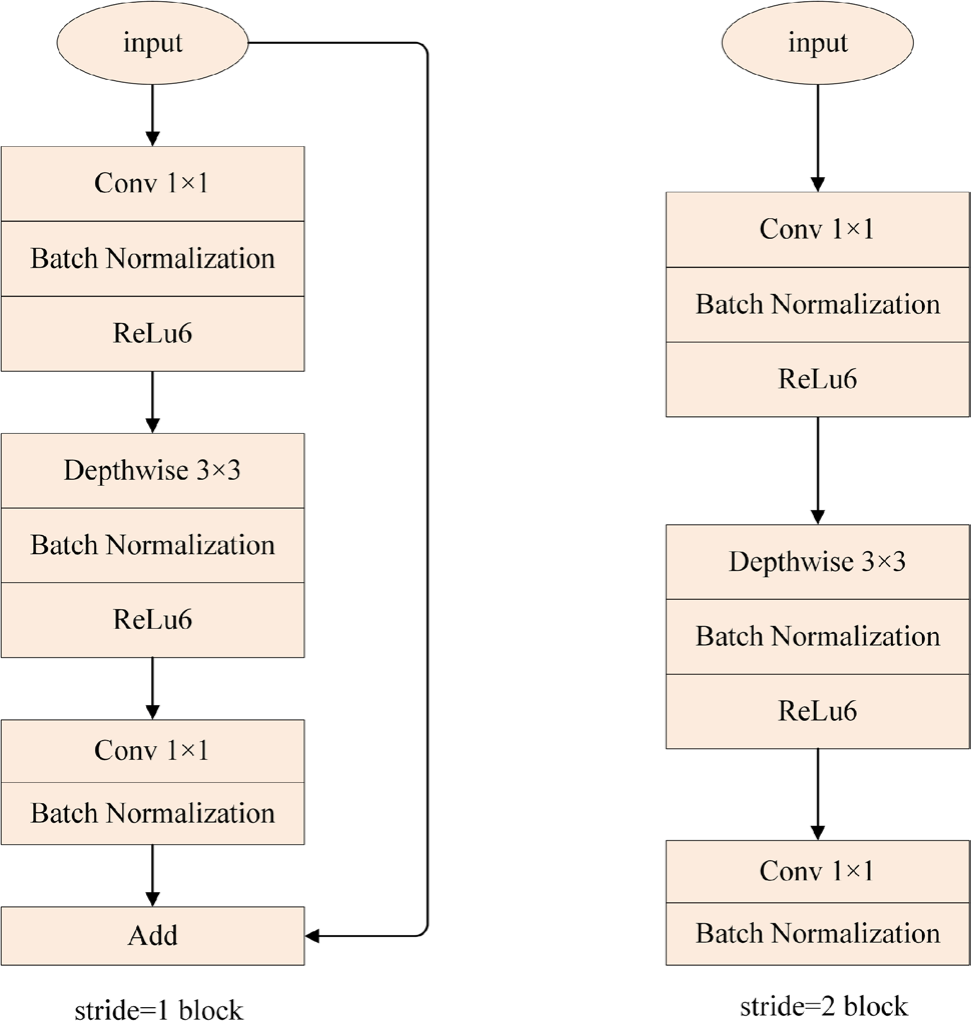
The structure of the Bottleneck layers.

The structure of the bottleneck layers with stride = 1 and stride = 2 is shown in Fig. 3. Each bottleneck block has three layers in turn. The first layer is a $1\times 1$ convolutional layer followed by a ReLU6 operation to expand the channels in the data. ReLU6 is the modification of rectified liner unit (ReLU). For the input x, ReLU and the ReLU6 are given as follows:

3 $$\begin{aligned} ReLU(x)= &   max(x,0) \end{aligned}$$

4 $$\begin{aligned} ReLU6(x)= &   min(max(x,0),6) \end{aligned}$$

The second layer contains $3\times 3$ filters which performs depth wise separable convolution operation. It extracts the features from the input and passes it to the next layer. The third layer known as a projection layer which reduces the number of channels is a $1\times 1$ convolutional layer without ReLU operation.

Another crucial feature of MobileNetV2 is the presence of residual connections, which are also known as skip connections or shortcut connections. The residual connections provide an alternative path for the gradient to flow during both forward and backward propagation. During backpropagation, the presence of these skip connections allows the gradient to flow directly through the network, reaching earlier layers without being significantly attenuated. The gradient flow through the skip connections helps to alleviate the vanishing gradient problem that often occurs in deep networks. By ensuring that the gradient can propagate effectively to earlier layers, the weights of those layers can be updated more efficiently during the training process.

The bottleneck blocks and corresponding input size of MobileNetV2 architecture are described in Table 1, where t represents the expansion factor, c represents the number of output channels, n represents the repeating number of blocks and s indicates the strides.

**Table 1:** Structure of MobileNetV2.

Input	operater	t	c	n	s
$224^2\times 3$	conv $3\times 3$	–	32	2	2
$112^2\times 32$	Bottleneck	1	16	1	1
$112^2\times 16$	Bottleneck	6	24	2	2
$56^2\times 24$	Bottleneck	6	32	3	2
$28^2\times 32$	Bottleneck	6	64	4	2
$14^2\times 64$	Bottleneck	6	96	3	1
$14^2\times 96$	Bottleneck	6	160	3	2
$7^2\times 160$	Bottleneck	6	320	1	1
$7^2\times 320$	conv2d $1\times 1$	–	1280	1	1
$7^2\times 1280$	avgpool $7\times 7$	–	–	1	–
$1\times 1\times 24$	conv2d $1\times 1$	–	k	–	

### Decoder

U-shape designed by UNet^62^ is a commonly used and simple end-to-end architecture. We used the variants of UNet in the medical image segmentation^63–66^ and grading^67^ and obtained good performace. In fact, efficiently extracting various features from the encoder for change detection task is broadly similar to do as image segmentation. Therefore, the decoder of UNet is introduced to detect the change of the images with the change feature gradually fused from the high layer to the low layer.

**Fig. 4:**
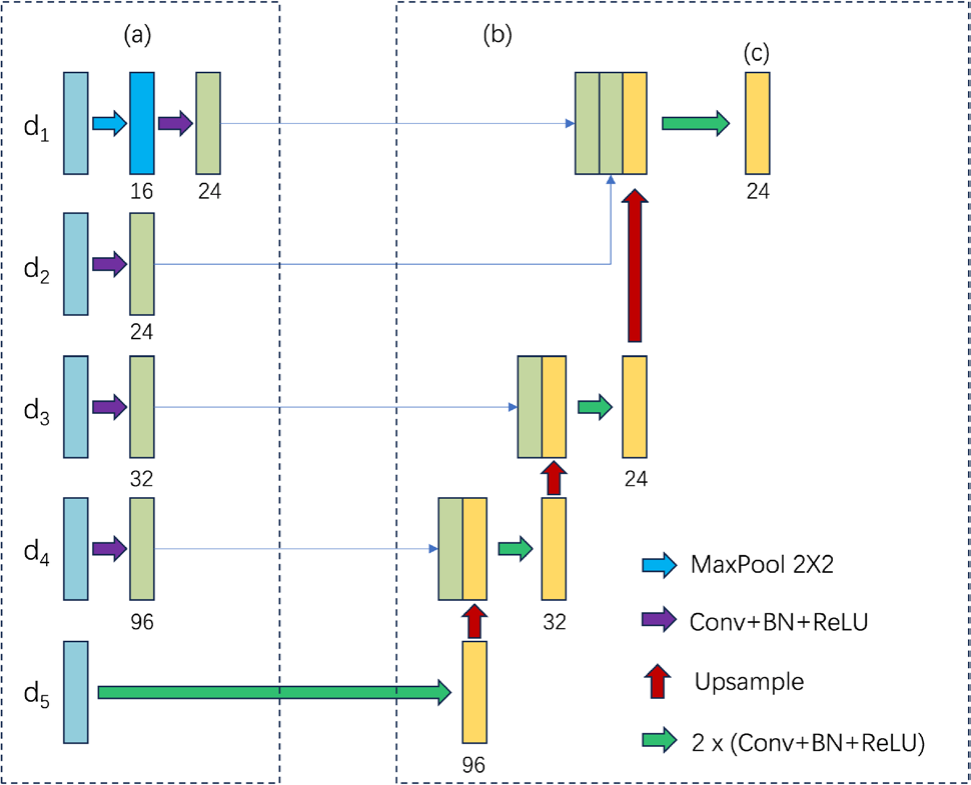
Core structure of the feature fusion module. (**a**) The backbone feature extraction. (**b**) The enhanced feature extraction network. (**c**) The input F to the prediction head.

As shown in Fig. 4a, it is referred to as the backbone feature extraction network. the difference image $d_i$ originates from elementwise subtraction operation between bitemporal remote sensing images at the *i*-th level ($i=1, 2,\ldots , 5$). Specifically, given the feature representations at two moments, $f_i^1$ and $f_i^2$, the elementwise subtraction operation based on Eq. (1) makes $d_i$ capture changes in features between different time points, highlighting variations in objects or scenes over time in RSCD. The hierarchical difference operation helps Mobile-CDNet understand and analyze dynamic changes in remote sensing images. We introduce additional max-pooling in the lowest-level difference image $d_1$ to alleviate noise. Since the low-level features include less semantic information and more pseudo-change ones, the lowest-level features are not integrated directly with higher-level ones. MaxPool with size $2\times 2$ is performed on it first to remove a certain amount of distractions, and then it is fused with the change features of the higher layer. Our Decoder experiments in the discussion section also show that this step is justified.

The enhanced feature extraction network is shown in Fig. 4b, We effectively integrate multi-level difference image features in the decoder to ensure robust performance from coarse to fine processing stages. The difference fusion module consists of ordinary convolutional layers and pooling layers, extracting differential information from backbone network features and enhancing the learning of useful information. For *i*-th level, the difference image $d_i$ is performed on convolution operations or MaxPooling and is feeded into the decoder and concatenated with the input upsampled high-level difference images to obtain the change feature maps. This paper introduces $d_i$ into an enhanced UNet network to enhance the accuracy and robustness of change detection. The highest level of difference image $d_5$ is performed convolution twice and then upsampled and concatenated with $d_4$ which has been operated by the convolution. Compared to the standard UNet decoder, our network includes the additional fusion of difference features from multiple levels, which is crucial for robust change detection across different resolutions. The decoder gradually fuses the difference images at different levels to reconstruct and restore the details and structure of the change images, which leverages the strengths of UNet in image segmentation and reconstruction and effectively capturing and representing change information in bitemporal remote sensing images.

The output of UNet is input in a pixel-wised classifier, and as shown in Fig. 4c, the input of the prediction head, is a low-resolution feature *F* used to generate the high-resolution probability map *P* by the introduced sigmoid function. The purpose of introducing the prediction head is to map the low-resolution feature to the high-resolution output to obtain accurate change area. Specifically, Eq. (2) serves as the mapping function, where the result *P* represents the likelihood of change in each pixel region.

### Loss function

For the change detection task, dealing with the class imbalance between changed and unchanged regions is a common challenge. In order to address this issue and enhance performance in complex scenes, a hybrid loss function is proposed which combines a binary cross-entropy (BCE) loss, denoted as $$ L_{bce}$$, and a dice (Dice) loss, denoted as $$ L_{dice}$$^68^. The BCE loss can be formulated as:

5 $$\begin{aligned} L_{bce}(c,g) = -[g\cdot logc +(1-g)\cdot \log (1-c)] \end{aligned}$$

where the dot product operation $(\cdot)$ denotes element-wise multiplication, c and g mean the predicted change map and the corresponding ground truth, respectively. The Dice loss is formulated as follows:

6 $$\begin{aligned} L_{dice}(c,g) = 1- \frac{2\cdot g\cdot c}{\Vert g\Vert +\Vert c\Vert } \end{aligned}$$

where $\Vert \cdot \Vert$ denotes $\ell _1$ the norm. The total loss is presented as:

7 $$\begin{aligned} L(c,g) =L_{bce}(c,g)+L_{dice}(c,g) \end{aligned}$$

By combining the BCE and dice losses, our hybrid loss function effectively addresses the class imbalance problem by capturing each distribution of the binary classification and the overlap regions between predicted and ground truth change masks. This combination facilitates robust and accurate detection of changes, particularly in scenarios with imbalanced change ratios and complex scenes.

## Results

### Dataset and experimental setup

**Fig. 5:**
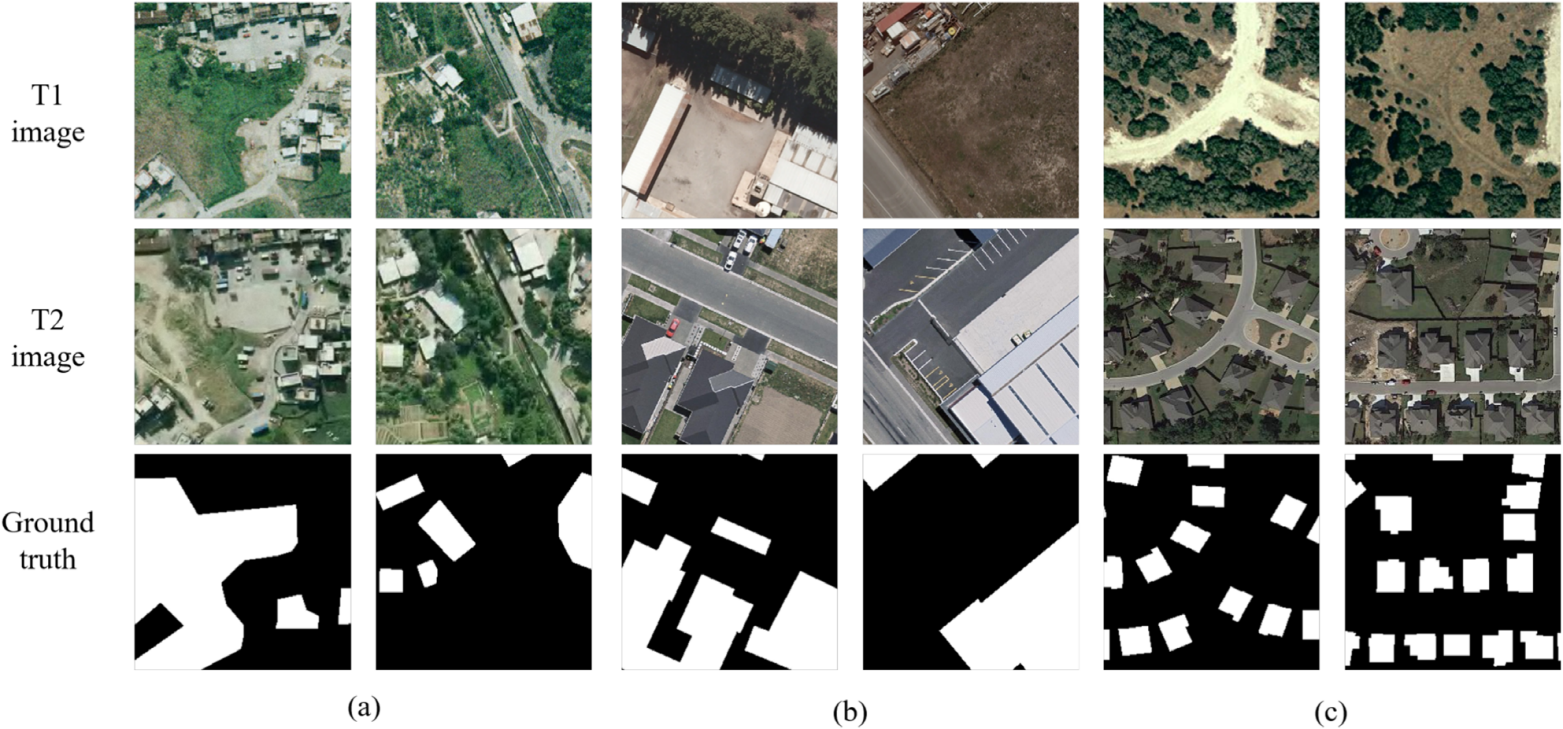
Bitemporal example images and ground truths from the datasets. (**a**) SYSU-CD. (**b**) BCDD. (**c**) LEVIR-CD.

Three extensive open-source, high-resolution remote sensing change detection datasets are introduced to validate the proposed method in this paper. Figure 5 showcases illustrative examples of these datasets.

Sun Yat-Sen University change detection (SYSU-CD)^54^ dataset consists of a comprehensive collection of 20,000 pairs of bitemporal remote sensing image patches. Each patch within the dataset exhibits a spatial size of $256\times 256$ pixels, offering a high spatial resolution of 0.5 meters. The dataset is carefully designed to capture a wide range of complex change scenes, such as road expansion, newly constructed urban buildings, changes in vegetation, suburban expansion, and groundwork prior to construction. The dataset follows an official split for training, validation, and testing, which is set at a ratio of 6:2:2. and we obtain a total of 12000/4000/4000 image pairs for training/validation/test, respectively.

Building change detection dataset (BCDD)^69^ focuses on an area that underwent significant changes due to a 6.3-magnitude earthquake in February 2011, followed by subsequent reconstruction efforts in the subsequent years. The dataset consists of aerial images captured in April 2012, covering a total area of 20.5 $km^2$ and encompassing 12,796 buildings (16,077 buildings in the 2016 dataset for the same area). These aerial images are of considerable size, measuring 32,507 $\times$ 15,354 pixels, and offer a high resolution of 0.075 meters. Following the methodology outlined in BIT^51^, we initially overlap crop images to create patches of size $256\times 256$ pixels. Consequently, we obtain a total of 6095/762/763 image pairs for training/validation/test, respectively.

Large-scale environmental vision instance recognition challenge dataset (LEVIR-CD)^70^ is meticulously crafted, consisting of a curated collection of 637 pairs of very high-resolution (VHR) Google Earth (GE) image patches. Each patch within the dataset boasts a spatial size of $1024\times 1024$ pixels, ensuring exceptional detail, and a resolution of 0.5m/pixel. These image pairs vividly depict bitemporal data captured over a time span ranging from 5 to 14 years, providing a comprehensive view of significant land-use changes. The dataset encompasses a diverse range of building types, including villa residences, tall apartments, small garages, and large warehouses. Following the official dataset split setting of 7:1:2, we overlap crop images to generate patches of size $256\times 256$ and obtain a total of 7120/1024/2048 image pairs for training/validation/test, respectively.

This paper builds a framework based on PyTorch, and all experiments are implemented based on NVIDIA RTX 2080Ti GPU (11G) and Python 3.8.0. We choose Adam^71^ as the optimizer to train the network, and the batch size is set to 16. The number of iterations of the network is 200 epochs. The initial learning rate of the network is 5e-4, which drops to 1/10 of the original every 100 epochs.

### Evaluation metrics

We employ a set of widely used evaluation metrics^72^ to rigorously evaluate the performance of our RSCD model. These metrics, namely the Kappa coefficient ($\kappa$), intersection over union (IoU), F1-score (F1), recall (Rec), and precision (Pre), provide comprehensive insights into different aspects of the model’s performance. Here are the refined definitions of these evaluation metrics:

8 $$\begin{aligned} Pre= &   \frac{Tp}{Tp + Fp} \end{aligned}$$

9 $$\begin{aligned} Rec= &   \frac{Tp}{Tp + Fn} \end{aligned}$$

10 $$\begin{aligned} F1= &   \frac{2Pre\cdot Rec}{Pre + Rec} \end{aligned}$$

11 $$\begin{aligned} OA= &   \frac{Tp + Tn}{Tp + Fp + Tn + Fn} \end{aligned}$$

12 $$\begin{aligned} P_e= &   \frac{(Tp + Fn)\cdot (Tp + Fp)+(Fp + Tp)\cdot (Fn + Tp)}{(Tp + Fp + Tn + Fn)^2} \end{aligned}$$

13 $$\begin{aligned} \kappa= &   \frac{OA-P_e}{1- P_e} \end{aligned}$$

14 $$\begin{aligned} IoU= &   \frac{Tp}{Tp + Fp + Fn} \end{aligned}$$

where Tp, Fp, Tn, and Fn denote the numbers of true positives, false positives, true negatives, and false negatives, respectively. Additionally, we provide detailed information about the model parameters and computation costs of the aforementioned methods for reference. This information gives insights into the complexity and efficiency of the models used in the paper, allowing for comparison and analysis of their computational requirements.

### Compared methods

In order to prove the effectiveness of the model in this paper, it is compared and analyzed with the current mainstream network models. They are L-UNet^73^, FC-Siam-diff^47^, FC-Siam-conc^47^, IFNet^59^, SNUNet^49^, A2Net^74^ and BIT^51^, which contain four lightweight networks FC-Siam-diff32, FC-Siam-conc, A2Net and BIT. These approaches are representative of various techniques and architectures for bitemporal change detection, where different architectures and mechanisms to effectively capture temporal information are designed and fused to enhance the accuracy of change detection models. These methods are briefly described as follows. For a fair comparison, we run on the same environment and configuration with the same number of training epoches and loss function as ours.

L-UNet combines semantic segmentation and change detection using a fully convolutional LSTM network. By capturing the temporal relationship of feature vectors across all encoders, the model can effectively analyze the changes over time. FC-Siam-diff utilizes a Siamese fully convolutional network (FCN) to extract multi-level features from the bitemporal images. Then, it computes the feature difference to fuse the information from two phases, enabling effective change detection. FC-Siam-conc takes a Siamese FCN to extract multi-level features. However, instead of computing feature differences, it combines the features through feature concatenation, thereby incorporating two phases to detect change. IFNet utilizes a multi-scale feature stitching technique through applying channel and spatial attention to the bitemporal features at various levels of the decoder. Deep supervision is employed to train intermediate layers to improve the model’s performance. SNUNet combines Siamese networks and NestedUNet to extract high-level features from the images. Channel attention is applied to the features at each stage of the decoder to enhance the recognition of important features. Deep monitoring can also be employed to further refine intermediate features. A2Net incorporates progressive feature fusion and a supervised attention mechanism. The NAM module enhances feature representation, the PCIM module extracts temporal difference information, and the SAM module reweights features, enabling efficient change detection. BIT inputs the convolved bitemporal image with markers into a transformer encoder to model context in the marker-based space-time. The learned context-enriched labels are then fed back to the pixel space to refine the original features through the decoder.

### Result

#### SYSU-CD

Table 2 lists the statistical the quantitative accuracy and parameter quantities of the detection results of different methods on the SYSU-CD dataset. When dealing with the complex scene as SYSU-CD, the proposed method in this paper is superior to other models except A2Net in the four indicators of $\kappa,$ IoU, F1, and Rec of the changing foreground category. This advantage may be attributed to the self-attention module integrated in the decoder of A2Net, which improves its ability to capture diverse changes, particularly for datasets like SYSU-CD that include multiple types of scene variations. Especially in the recall rate, the proposed method is 0.95% higher than the suboptimal A2Net, indicating that Mobile-CDNet can more accurately identify changing building objects. In terms of suppressing false changes, the Kappa coefficient of Mobile-CDNet is as high as 77.51%, indicating that the detection results of Mobile-CDNet are more consistent with the real labels than other methods except A2Net, which confirms that the method proposed in this paper is effective in multi-temporal high-resolution remote sensing image. Compared with other models, Mobile-CDNet achieves the best detection results with the least amount of calculation. Although FC-Siam-diff and FC-Siam-conc have fewer parameters, the overly simple network structure affects its detection performance. As far as BIT is concerned, both methods have the similar parameters, but Mobile-CDNet obtains a higher level of detection performance. As for A2Net, it outperforms Mobile-CDNet in $\kappa ,$ IoU, and F1, but Mobile-CDNet has fewer parameters.

**Fig. 6:**
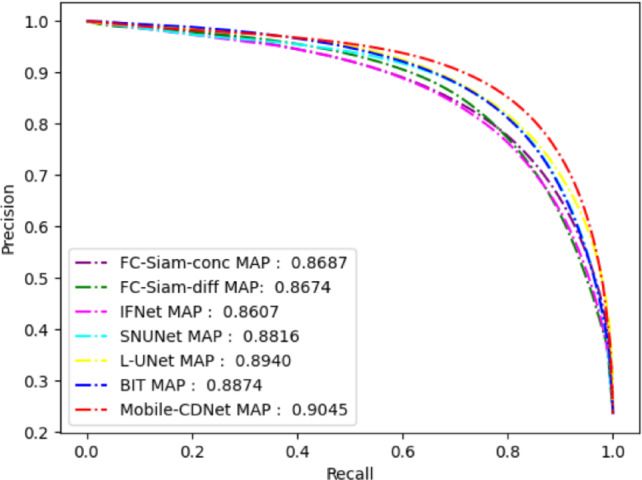
P-R curves of different models on SYSU-CD.

The PR curve is often used to evaluate the performance of classification algorithms. It is drawn based on the precision and recall of the model under different classification thresholds, which can more objectively evaluate the classification performance of the classifier. For the change detection task, the PR curve focuses on the change foreground target, which represents the trade-off relationship between the precision and recall rate of the change target in the detection results. Figure 6 shows the precision-recall curve (hereinafter referred to as PR curve) of Mobile-CDNet compared the involved methods on SYSU-CD. The closer the PR curve to the upper right is, the better the separability of the range map predicted by the model is, which also means the stronger the detection performance of the model. From Fig. 6, it can be seen that the PR curve of Mobile-CDNet is closest to the upper right, indicating that Mobile-CDNet is superior to the other methods in the robustness of highlighted change areas and the suppression of false changes, as well as the sensitivity to classification thresholds.

**Fig. 7:**
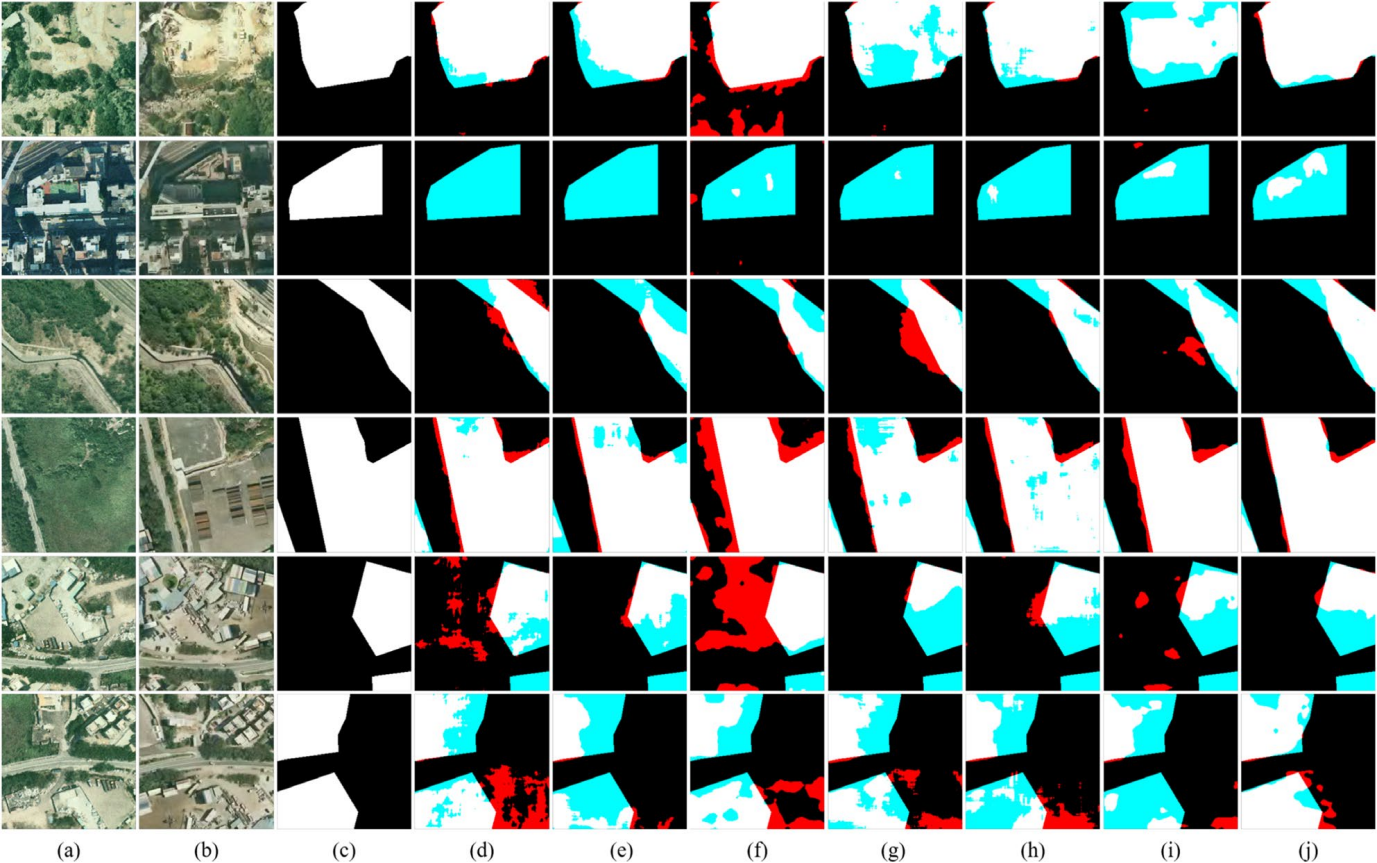
Visual comparisons on SYSU-CD. (**a**) t1 images. (**b**) t2 images. (**c**) Ground truth. (**d**) FC-siam-conc. (**e**) FC-siam-diff. (**f**) IFNet. (**g**) SNUNet. (**h**) L-Unet. (**i**) BIT. (**j**) Mobile-CDNet. The rendered colors represent true positives (white), false positives (red), true negatives (black), and false negatives (blue).

Figure 7 shows a visual example of the change detection results of different methods on SYSU-CD, where black represents the background. It can be intuitively seen that the proposed method performs best among several compared methods for changes that are not easy to identify. As shown in Fig. 7j, Mobile-CDNet has fewer missed regions and false ones than other models.

**Table 2:**
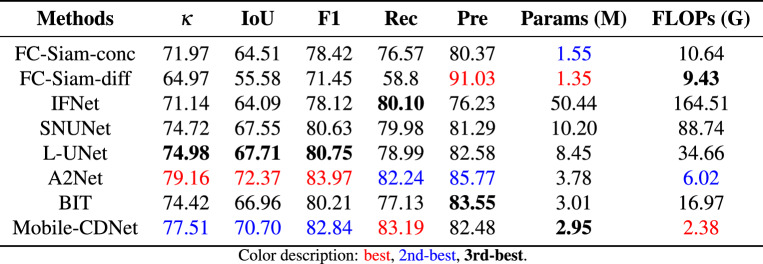
Quantitative comparison on SYSU-CD.

#### BCDD

Table 3 lists the statistical the quantitative accuracy and parameter quantity of the detection results of different methods on BCDD. It can be seen that when dealing with the highly similar scene as BCDD, the proposed method is superior to other models except A2Net in the four indicators of $\kappa,$IoU, F1, and Rec of the changing foreground category. In terms of suppressing false changes, the Kappa coefficient of Mobile-CDNet is as high as 94.27%, indicating that the detection results of Mobile-CDNet are more consistent with real labels than the other methods. For effectiveness on the change detection problem, compared with other models, Mobile-CDNet achieves the highest detection accuracy with the least amount of calculation. Although FC-Siam-diff has the least number of parameters, the overly simple architecture affects its detection performance. Compared with the BIT method close on the number of parameters, the detection performance of Mobile-CDNet reaches a higher level. While A2Net achieves better performance than Mobile-CDNet in $\kappa,$ IoU, and F1, Mobile-CDNet stands out with a smaller number of parameters.

**Fig. 8:**
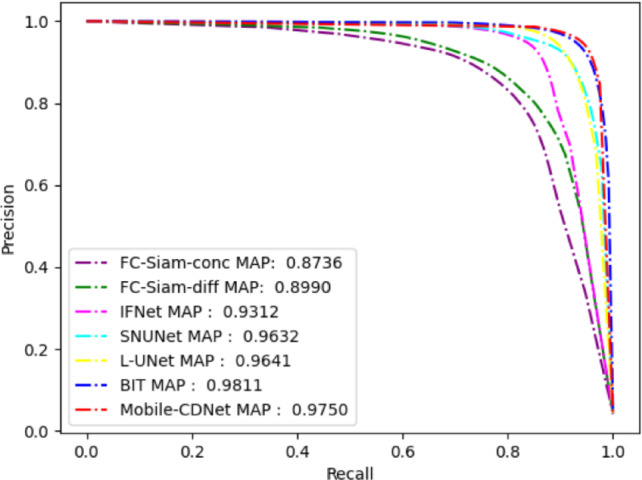
P-R curves of different models on BCDD.

Figure 8 shows the PR curve results of Mobile-CDNet and the methods involved in the comparison on the BCDD dataset. The PR curve of Mobile-CDNet is closest to the upper right, indicating that Mobile-CDNet is superior to the other methods in the robustness of highlighted change areas and the suppression of false changes, as well as the sensitivity to classification thresholds.

**Fig. 9:**
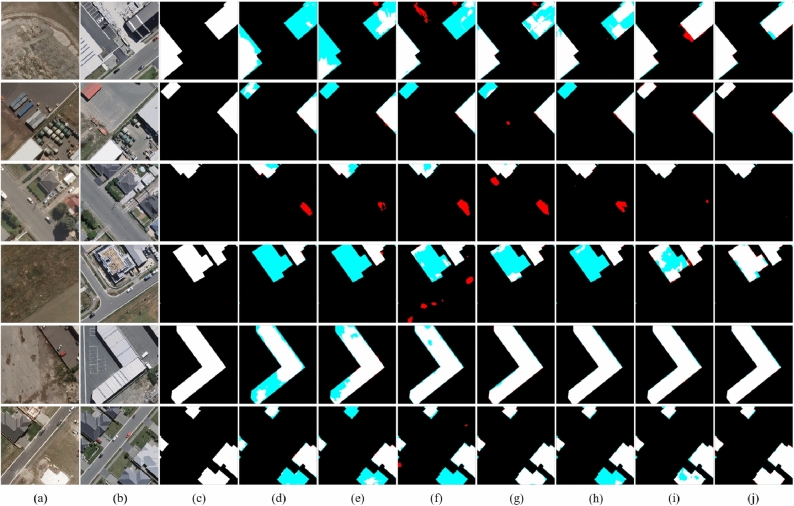
Visual comparisons on BCDD. (**a**) t1 images. (**b**) t2 images. (**c**) Ground truth. (**d**) FC-siam-conc. (**e**) FC-siam-diff. (**f**) IFNet. (**g**) SNUNet. (**h**) L-Unet. (**i**) BIT. (**j**) Mobile-CDNet. The rendered colors represent true positives (white), false positives (red), true negatives (black), and false negatives (blue).

Figure 9 shows a visual example on BCDD. It can be intuitively seen that the proposed method performs the best among several building change detection methods, whether the different scales of changing buildings or the removal of distraction of spurious changes. The detection results of Mobile-CDNet are the most consistent with the reference labels, and there are no obvious missed regions and false ones. However, as shown in the first and second rows of Fig. 9, for the detection results of FC-diam-diff, IFNet, SNUNet and L-UNet, individual changing building is not completely detected, which brings a lot of missed change regions. The third and fourth rows of Fig. 9 presents the complex urban scenes, and their detection results on individual confusing and unchanged building objects are detected incorrectly. The proposed method overcomes the distractions of noise well, and the detection results are neat and clean, which shows that Mobile-CDNet has better robustness on BCDD.

**Table 3:**
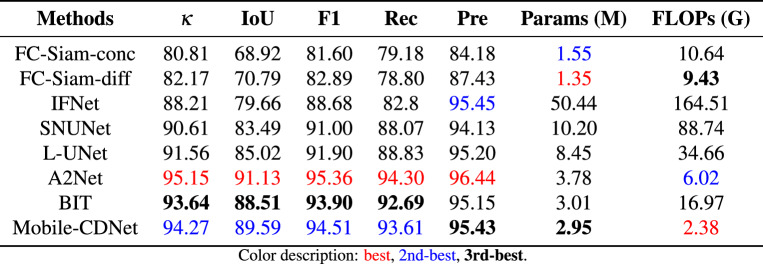
Quantitative comparisons on BCDD.

#### LEVIR-CD

The quantitative detection accuracy of different methods on the LEVIR-CD dataset is presented in Table 4. It can be observed that there remains a slight gap between the performance of Mobile-CDNet and that of the best-performing network, A2Net, in terms of the F1-score. Specifically, Mobile-CDNet achieves an F1-score of 90.89%, which is slightly lower than the 91.28% achieved by IFNet, despite the fact that IFNet involves 50.44 M parameters. Precision-wise, Mobile-CDNet is also 1.52% lower than IFNet. While A2Net also shows strong performance, it, like IFNet, relies on a significantly more complex architecture.

This performance gap can largely be attributed to a deliberate trade-off between detection accuracy and computational efficiency. IFNet has approximately 17 times more parameters and 69 times more FLOPs than Mobile-CDNet, making it less suitable for real-world applications with limited computational resources, such as UAVs and satellite platforms. To address the potential degradation in accuracy due to the lightweight design, we introduce three key modules to maintain robust performance. The first is an enhanced MobileNetV2 backbone that is responsible for extracting essential features. The second is the adoption of depthwise separable convolutions, which effectively reduce computational overhead while preserving feature representation capacity. The third is a difference fusion module, which improves the ability of network to capture and emphasize salient change information.

**Fig. 10:**
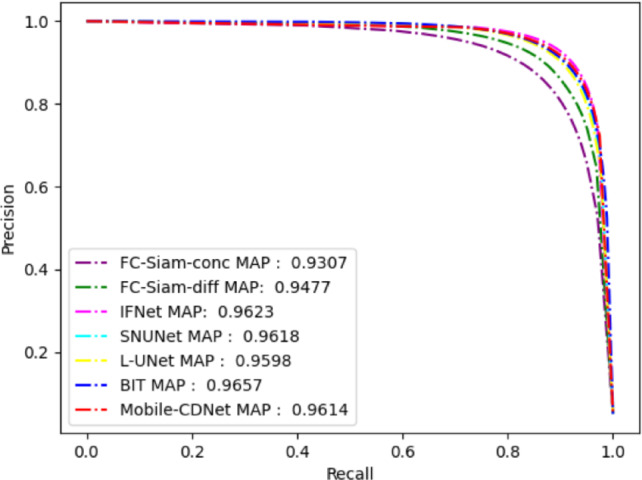
P-R curves of different models on LEVIR-CD.

**Fig. 11:**
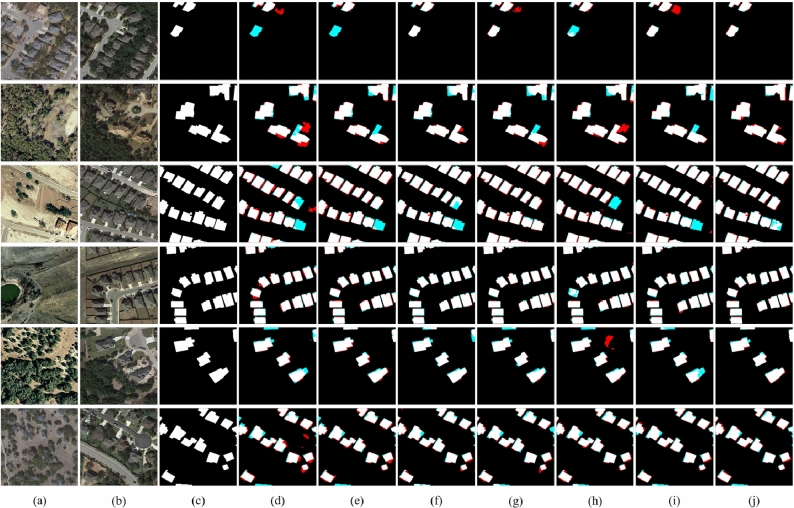
Visual comparisons on LEVIR-CD. (**a**) t1 images. (**b**) t2 images. (**c**) Ground truth. (**d**) FC-siam-conc. (**e**) FC-siam-diff. (**f**) IFNet. (**g**) SNUNet. (**h**) L-Unet. (**i**) BIT. (**j**) Mobile-CDNet. The rendered colors represent true positives (white), false positives (red), true negatives (black), and false negatives (blue).

As shown in the precision-recall curve in Fig. 10, Mobile-CDNet is positioned relatively close to the upper-right corner, which indicates strong robustness on the LEVIR-CD dataset. Additionally, Fig. 11 provides visual examples of detection results. Although Mobile-CDNet demonstrates a slight performance gap in numerical metrics compared with IFNet, it produces cleaner and more coherent outputs, especially on small-scale building targets. Furthermore, it generates fewer false detections than other methods, which verifies the effectiveness and practicality of Mobile-CDNet in real-world change detection tasks.

**Table 4:**
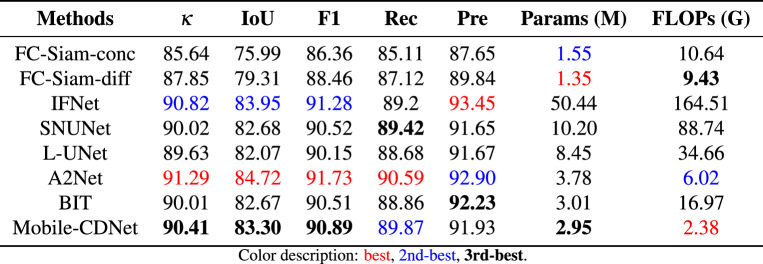
Quantitative comparisons on LEVIR-CD.

## Discussion

For encoder, we further validate the proposed method with ResNet18 (#01 in Table 5). From the results in Table 5, we note that the backbone with MobileNetV2 slightly outperforms that with ResNet18 on the LEVIR-CD dataset. As a result, the proposed methods with MobileNetV2 achieves satisfactory performance, indicating the effectiveness of the lightweight backbone.

We intend to validate the effectiveness of adding max-pooling to the lowest layer of difference feature to remove distractions in decoder. The UNet decoder is taken to fuse the different levels of the change features. Additionally, maximizing the pooling of the lowest-level difference features is designed and fused with the second-stage difference features at the same resolution. A comparison is made between the two fusion methods (#03 in Table 5), and the results shown in Table 5 demonstrate that the our method obtains a better fusion effect after maximizing the lowest layer difference features.

The hybrid loss consisting of the Dice loss and BCE loss is adopted in this paper. To validate the reasonability of the loss function, we conduct experiments on the methods with only BCE loss or only Dice loss (#05 in Table 5), and report the quantitative comparisons results in Table 5. The method with Dice loss performs better than that with BCE loss. Besides, only using Dice loss or BCE loss has worse detection performance than the hybrid loss. Therefore, combining the hybrid of the Dice loss and BCE loss in our model is a better choice.

**Table 5:** Quantitative comparisons of the proposed method with various settings on RSCD datasets.

No.	Variants	Efficiency		Evaluation metrics				
		Params (M)	FLOPs (G)	$\kappa$	IoU	F1	Rec	Pre
(a) Different Backbones on LEVIR-CD								
#01	ResNet18	15.60	60.19	90.36	83.02	90.84	89.54	**92.19**
#02	MobileNetV2	**2.95**	**2.38**	**90.41**	**83.30**	**90.89**	**89.87**	91.93
(b) Different Decoders on LEVIR-CD								
#03	UNet-Decoder	**2.92**	2.43	90.23	83.02	90.72	89.08	**92.42**
#04	Our-Decoder	2.95	**2.38**	**90.41**	**83.30**	**90.89**	**89.87**	91.93
(c) Different Losses on LEVIR-CD								
#05	BCE	2.95	2.38	89.82	82.36	90.33	88.17	**92.60**
#06	Dice	2.95	2.38	90.21	82.98	90.70	89.27	92.17
#07	BCE+Dice	2.95	2.38	**90.41**	**83.30**	**90.89**	**89.87**	91.93
(d) EEF on LEVIR-CD								
#08	w/o EEF	**2.95**	**2.38**	90.41	83.30	90.89	89.87	91.93
#09	w/ EEF	3.18	3.12	**90.59**	**83.60**	**91.07**	**90.04**	**92.12**
(e) MO on BCDD								
#10	w/o MO	2.95	2.38	94.27	89.59	94.51	93.61	95.43
#11	w/ MO	2.95	2.38	**94.87**	**90.63**	**95.08**	**93.83**	**96.37**

Significant values are in bold.

In this paper, the problem of change detection in remote sensing images at two phases is analyzed, and a lightweight change detection algorithm is developed to deal with the existing problems. Our method has significantly improved the performance of change detection, but there are still some problems to be further researched. According to current experiment results, we will explore and analyze from the following aspects: (1) Although the method proposed in this paper has a low amount of calculation and parameters, the accuracy of small target change detection is needed to be improved such as the results on LEVIR-CD. This may be related to the fact that we have adopted the lightweight backbone where the feature extraction is not so sufficient. Before the differential operation of the encoder, we use FPN^75^ to fuse the five-level features of each time phase extracted by MobileNetV2 from the high level to the low, and enhance the feature representation (#08 in Table 5). The results on LEVIR-CD are shown in Table 5, where the enhanced backbone, powered by enhanced encoder features (EEF), is superior to Mobile-CDNet in the five indicators of $\kappa,$ IoU, F1, and Rec of the changing foreground category. Especially in the $\kappa,$ the proposed method is 0.68% higher than the suboptimal Mobile-CDNet which indicates that Mobile-CDNet can more accurately detect changing buildings. It can be seen that in the encoder stage, the enhanced features extracted by the backbone will improve the accuracy of the final change detection results. Therefore, in future work, we should further focus on designing the backbone to fuse the features and improve the detection accuracy while containing the reduction of algorithm on parameters and computation. (2) At present, many networks add loss functions to the outputs of different levels in the decoder to optimize the architecture when backpropagating. Following this line of thought, we have added losses to the first three outputs of the decoder, with multiple outputs (MO) from coarse to fine (#10 in Table 5), which is validated on BCDD and the results are shown in Table 5. It can be seen $\kappa,$ IoU, F1, and Rec are all improved compared with the former, and the amount of parameters and calculations are basically unchanged. It should be noticed that F1 increased by 0.57%. Hence this operation may be helpful to improve network performance, and in future work, we will study on different losses for the output of different stages of the decoder to further improve our method.

## Conclusion

As it is difficult to adapt to some constrained devices with hardware and software such as satellites for RSCD and most of architectures have a large amount of parameters and calculations, the lightweight network Mobile-CDNet is designed in this paper. In order to reduce the computing costs and the inference time, MobileNetV2 is taken as the backbone to extract bitemporal image features and then obtains the change features at different stages through simple differential operations. Further, the UNet decoder is taken and improved to enhance the change features by gradually integrating the features of each stage. Finally, we evaluate the model on three public datasets SYSU-CD, BCDD, LEVIR-CD, where our method obtains the F1 indicators 82.84%, 94.51% and 90.89% respectively with parameters 2.95M and computation 2.38G.

Compared with several mainstream networks, Mobile-CDNet greatly reduces the computational cost without sacrificing the accuracy of the architecture, and most of the evaluation indicators are better than the compared models. A great balance has been made between accuracy and performance. The PR curve and the visualized results also intuitively illustrate the superior performance of our method. In addition, the discussion shows that our network will have a lot of room for improvement in accuracy. For the future work, we have made preliminary explorations in enhancing the features of the backbone and adding multiple losses to the output of the decoder.

## Data Availability

The data that support the findings of this study are openly available, and three public datasets SYSU-CD, BCDD and LEVIR-CD are used in this paper. They can be obtained through https://github.com/liumency/SYSU-CD, https://gpcv.whu.edu.cn/data/building_dataset.html and https://github.com/justchenhao/LEVIR, correspondingly.

## Abbreviations

RSCD: Remote sensing change detection
CVA: Change vector analysis
PCA: Principal component analysis
MAD: Multivariate alteration detection
IR-MAD: Iterative reweighted multivariate alteration
KSFA: Slow feature analysis
CNN: Convolutional neural network
CV: Computer vision
AE: Autoencoder
RNN: Recurrent neural network
GAN: Generative adversarial network
LSTM: Long short term memory
VGG: Very deep convolutional networks for large-scale image recognition
SYSU-CD: Sun Yat-Sen University change detection
BCDD: Building change detection dataset
LEVIR: LEarning, VIsion and Remote sensing laboratory
FC-Siam-conc: Fully convolutional Siamese-concatenation
FC-Siam-diff: Fully convolutional Siamese-difference
BCE: Binary cross-entropy
